# Neural correlates of learning and trajectory planning in the posterior parietal cortex

**DOI:** 10.3389/fnint.2013.00039

**Published:** 2013-05-17

**Authors:** Elizabeth B. Torres, Rodrigo Quian Quiroga, He Cui, Christopher A. Buneo

**Affiliations:** ^1^Psychology Department, Cognitive Science, Computer Science, Sensory Motor Integration, Rutgers UniversityNew Brunswick, NJ, USA; ^2^Neurology, School of Medicine, Indiana UniversityBloomington, IN, USA; ^3^Department of Engineering, University of LeicesterLeicester, UK; ^4^Department of Psychiatry and Behavior Health, Brain and Behavior Discovery Institute, Medical College of Georgia, Georgia Regents UniversityAtlanta, GA, USA; ^5^School of Biological and Health Systems Engineering, Arizona State UniversityTempe, AZ, USA

**Keywords:** posterior parietal cortex, obstacle avoidance, reaching, planning, postural control

## Abstract

The posterior parietal cortex (PPC) is thought to play an important role in the planning of visually-guided reaching movements. However, the relative roles of the various subdivisions of the PPC in this function are still poorly understood. For example, studies of dorsal area 5 point to a representation of reaches in both extrinsic (endpoint) and intrinsic (joint or muscle) coordinates, as evidenced by partial changes in preferred directions and positional discharge with changes in arm posture. In contrast, recent findings suggest that the adjacent medial intraparietal area (MIP) is involved in more abstract representations, e.g., encoding reach target in visual coordinates. Such a representation is suitable for planning reach trajectories involving shortest distance paths to targets straight ahead. However, it is currently unclear how MIP contributes to the planning of other types of trajectories, including those with various degrees of curvature. Such curved trajectories recruit different joint excursions and might help us address whether their representation in the PPC is purely in extrinsic coordinates or in intrinsic ones as well. Here we investigated the role of the PPC in these processes during an obstacle avoidance task for which the animals had not been explicitly trained. We found that PPC planning activity was predictive of both the spatial and temporal aspects of upcoming trajectories. The same PPC neurons predicted the upcoming trajectory in both endpoint and joint coordinates. The predictive power of these neurons remained stable and accurate despite concomitant motor learning across task conditions. These findings suggest the role of the PPC can be extended from specifying abstract movement goals to expressing these plans as corresponding trajectories in both endpoint and joint coordinates. Thus, the PPC appears to contribute to reach planning and approach-avoidance arm motions at multiple levels of representation.

## Introduction

The process of planning reaching movements to visual stimuli begins with an image on the two retinas and ends with a complex spatiotemporal pattern of arm muscle activations. Understanding the intervening stages in this process remains one of the primary challenges of motor neuroscience. At an early sensory processing stage, the perceived target position is compared with the position of the limb, resulting in a desired “displacement vector” (Flanders et al., 1992; McIntyre et al., 1998; Crawford et al., 2004; Shadmehr and Wise, 2005; Buneo and Soechting, 2009). Subsequent computations require converting this abstract task-level representation of movement into a spatially and temporally organized sequence of hand positions required to achieve the reach goal, a process termed “trajectory formation” (Hoff and Arbib, 1993; Torres and Zipser, 2002). This mapping is non-trivial as there are an infinite number of possible hand paths consistent between two points in 3D space. Further computations are required to convert handpaths into joint angles and joint angles into joint torques and/or muscle activations. These latter transformations are also highly non-linear and understanding them will require advances in computational methods as well as insights gained through neurophysiological investigations of the brain areas involved in visually-guided reaching.

Although the distinct computations involved in transforming desired hand displacements into arm postures do not appear to map uniquely onto specific areas of the primate brain, neurophysiological studies in non-human primates have nonetheless succeeded in identifying neural correlates of some aspects of the movement planning process. Planning activity is best revealed during delayed response tasks, particularly memory-guided ones (Hikosaka and Wurtz, 1983), as these tasks temporally dissociate planning activity from both the sensory signals used to cue movements and the processes involved in movement execution, including reference. In arm movement related areas of the frontal lobe such as the motor cortex (M1), dorsal premotor cortex (PMd) and the supplementary motor area (SMA), these paradigms have revealed evidence for planning of high level kinematic parameters such as movement direction, amplitude, and speed etc., (Kurata and Wise, 1988; Shen and Alexander, 1997; Moran and Schwartz, 1999; Crammond and Kalaska, 2000; Messier and Kalaska, 2000; Churchland et al., 2006). Evidence for planning of dynamic (kinetic) parameters has also been identified in M1, PMd and SMA (Li et al., 2001; Padoa-Schioppa et al., 2002, 2004; Xiao et al., 2006).

The parietal cortex is also active during memory-guided reach and saccade tasks (Andersen and Buneo, 2002). Analyses of neural responses in non-human primates suggest that memory activity in the posterior parietal cortex (PPC) reflects movement *planning* and not simply a memory of previous sensory events or attention-related phenomena (Quian Quiroga et al., 2006; Cui and Andersen, 2007). Other studies have shown that reach planning activity in the PPC reflects the encoding of high level parameters of movement. For example, activity in MIP and area 5d (as well as PMd) appears to be consistent with the transforming of eye-centered information about targets and hand positions into a hand-centered movement vector (Batista et al., 1999; Buneo et al., 2002, 2008; Buneo and Andersen, 2006; Pesaran et al., 2006). Recent TMS, imaging, and clinical studies in humans are consistent with this view (Beurze et al., 2006, 2007, 2010; Khan et al., 2007; Vesia et al., 2008).

Studies in both humans and monkeys have provided evidence that frontal and parietal reach-related areas are involved in encoding not only abstract kinematic parameters but also detailed movement trajectories (Georgopoulos et al., 1983, 1988; Hocherman and Wise, 1991; Schwartz, 1994; Desmurget et al., 1999; Serruya et al., 2002; Taylor et al., 2002; Carmena et al., 2003; Diedrichsen et al., 2005; Mulliken et al., 2008a; Archambault et al., 2009). However, with one notable exception (Hocherman and Wise, 1991) these studies did not employ delayed response tasks requiring the performance of different trajectories between identical starting and goal locations. As a result it is unclear the extent to which these areas are involved in trajectory *planning*, as opposed to trajectory *execution.* In addition, since evidence for trajectory execution comes primarily from the study of overlearned reaches, it remains largely unexplored whether the parieto-frontal reach network also participates in the formation of new trajectories. This would require spontaneously evoking—i.e., without explicit visual or other cues (Hocherman and Wise, 1991)—curved trajectories and comparing memory activity for the same starting position and target position but different movement paths. Furthermore areas involved in the planning of new trajectories could provide a reliable learning signal appropriate for calibrating internal models of arm dynamics and could also provide a real-time kinematic signal for brain-machine interfaces.

The study of curved trajectories is important from a computational standpoint as well. Curved trajectories are also shortest-distance “straight” paths in non-Euclidean geometries (Gray, 1998). As such they can be conceived as generalized solutions to distance-related optimization problems for which the Euclidean straight reaching trajectories, which have been the main focus of research, are only a particular case (Torres and Zipser, 2002; Torres and Andersen, 2006; Biess et al., 2011). In this sense the empirical study of curved trajectories and neural correlates of the kinematics may help us design better computational models with explanatory power on possible general brain solutions to the complex problems of trajectory formation, coordinate transformations and reference frames. Several aspects of these problems have been linked to areas in the PPC (Hauschild et al., 2012).

**Figure 1:**
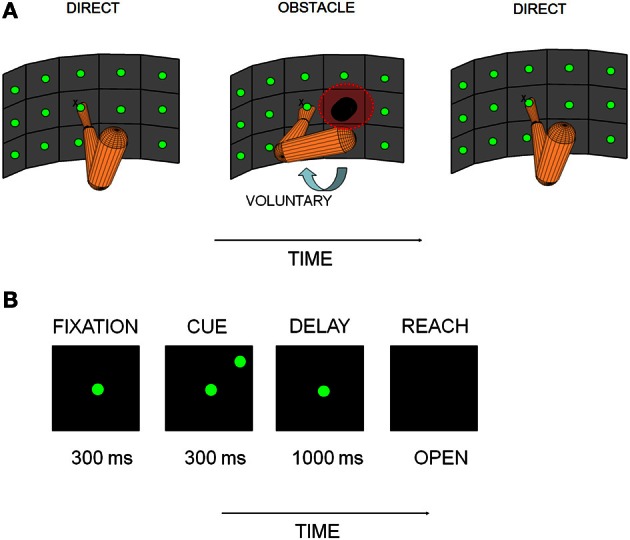
**Experimental apparatus and behavioral paradigm. (A)** Vertically oriented array of pushbuttons used to cue reaches is illustrated for each experimental block, along with a schematic representation of the starting posture of the arm. The posture change shown in the obstacle avoidance block (OA) represents an approximation of the anticipatory change in initial arm posture that the sensors registered. **(B)** Sequence of events on single trials. The experimental paradigm consisted of a baseline/fixation epoch (300 ms), followed by cue (300 ms), delay/memory (800–1000 ms) and reach epochs (variable duration as movement time was not controlled).

To examine potential roles for the PPC in adaptation to both spatial temporal aspects of new trajectory planning we recorded the activity of MIP neurons in a memory-guided obstacle avoidance task that the animals had to resolve and learn to implement in real time. This reaching task required different degrees of curvature and path lengths between the same starting and goal locations. The new movements also required adjustments of speed, a process that behavioral analyses have shown takes much longer to master once the spatial curve has been resolved (Torres and Andersen, 2006; Torres, 2010). Animals first planned and executed a block of direct (point-to-point) reaches between locations on a vertically oriented target array (Figure 1) and then attempted to move between the same locations in the presence of a physical obstacle, which required very different movement trajectories. The findings suggest that MIP plays a more extensive role in movement planning than previously thought, and is involved not only in specifying high level movement parameters, such as movement goals, but also in mapping these parameters into corresponding movement trajectories in both endpoint and joint/muscle coordinates.

## Materials and methods

All experimental procedures were conducted according to the “Principles of Laboratory Animal Care” (NIH publication no. 86–23, revised 1985) and were approved by the California Institute of Technology Institutional Animal Care and Use Committee.

### Behavioral paradigm

Two rhesus monkeys (W and C) were trained to perform a sequence of memory-guided reaching tasks in the dark. Reaches were made to targets on a three row by five column vertically oriented array of reach targets (Figure 1A). The reach targets were touch sensitive buttons that were 3.7 cm in diameter and set 7.5 cm apart (18°) on a vertically-oriented Plexiglas board. In order to actuate the buttons and move between positions on the board, the animals had to pull their hand away from the surface and then place it back again once the target was acquired. Due to the large range of target positions employed in this study, movements involved changes in both shoulder angle and, to a lesser extent, elbow angle. However, since targets were constrained to lie on a surface, limb movements involved minimal variation along the depth axis. Animals performed a minimum of 10 trials always from the central location of the board (the fixation point) to each peripheral target location. The number of targets ranged from 8 to 14, depending on the presence/absence of an obstacle and the performance level of the animals in a given session. In this experimental design, block refers to the experimental block (direct reaches D1; obstacle avoidance reaches OA; and direct reaches again D2). The word epoch refers to the different segments of relevance within a block (baseline, cue, memory and reach to be described later). The portion of a block is the grouping of the data for a subset of trials (10 minimum) from the total number of trials in that block, in the order in which they were acquired (e.g., the first 10 trials of a block or the last 10 trials).

Eye and hand movements were guided and monitored by a real-time behavioral control program written in LabVIEW and running on a real-time PXI platform (National Instruments Inc., TX). Eye fixation at the central location of the board was monitored using a scleral search coil in animal C and with an optical system in animal W (ISCAN Inc., MA, USA). Arm postures and hand trajectories were recorded with electromagnetic sensors (Polhemus Fastrak, 120 Hz) placed at the approximate positions of the shoulder, elbow and wrist and affixed to a primate jacket (Lomir Biomedical Inc.). A previously described algorithm was used to recover seven of the arm's joint angles that best reconstructed the hand, elbow and shoulder trajectories (Torres et al., 2011).

Animals performed a sequence of three blocks of trials as depicted in Figure 1A. An initial block of direct reaches (D1) was followed by a block of obstacle avoidance reaches (OA) and then by another block of direct reaches (D2) identical to D1. The animals had extensive training for the direct reaches, however, prior to the start of our recordings they had no previous training in the OA reaches. Thus each day the only exposure that they had to this aspect of the paradigm was during the experiments. This enabled us to measure the cells' activities in real time both during the learning/adaptation period of the OA block and also during the de-adaptation to the OA reaches (i.e., in D2).

#### Initial block of direct reaches (D1)

In the initial block of trials (D1), animals were required to reach directly from a central button to a remembered peripheral target location. The timing of the behavioral events is shown in Figure 1B. At the beginning of each trial, animals had to fixate straight ahead while holding the central button for 300 ms (FIXATION epoch). A target light in the periphery was then flashed for 300 ms (CUE) followed by a variable delay period (chosen across trials from a Gaussian distribution) between 800–1000 ms where the animals had to withhold the reach (MEMORY) and maintain fixation until the extinction of the fixation light (GO signal). Then, the animals moved the hand to the target while continuing to maintain fixation (REACH). Movement duration was not controlled in this experiment. Trials were aborted if the animal broke fixation, the animal did not initiate the reach within 1500 ms of the GO signal, or if the target was missed.

#### Obstacle avoidance block (OA)

The sequence and timing of behavioral events in OA was identical to that in D1. Upon completion of D1 the preferred location of the cell during the memory epoch was determined by calculating the mean firing rate at each location of the board and identifying the location for which the cell fired maximally. This preferred location was used to guide the placement of the obstacle. The obstacle was placed whenever possible inside the memory field of the neuron or at the nearest possible board location in cases where the constraints of the board did not allow placement inside the field.

Prior to the initiation of the OA block, the lights in the room were turned ON such that the animals saw the application of the obstacle. The obstacles consisted of black cylinders that, when attached to a pushbutton, protruded 4 inches out of the board. The animals could see their location in full light but once the recording session started and the lights went off, the animals could not see the obstacles. As a result the animals had to memorize the locations of the obstacles in order to avoid them and reach the target. This required a change not only in the initial posture of the arm but also a rotation of the initial movement direction in three dimensions, leading to reaches of longer length and distinct curvature from those in D1 (Torres, 2010).

#### Second block of direct reaches (D2)

The obstacle block was followed by another block of direct reaches (D2). Prior to the initiation of D2, the lights in the room were turned on so that the animals saw the removal of the obstacle. The light was then turned off so that the animals performed the D2 block in complete darkness as in the D1 block.

### Control experiment to examine posture-related responses

At the start of the OA block the arm sensors revealed that both animals voluntarily adjusted their initial arm postures in anticipation of the new block of curved reaches (illustrated schematically in Figure 1A). As a result, we used a control experiment to distinguish changes in firing rate due to differences in initial arm posture (Scott et al., 1997) from changes due to differences in planned trajectories. In this experiment a custom-built apparatus was attached to the primate chair that enforced a particular initial arm posture without interfering with the reaches, which were performed in the absence of an obstacle. This apparatus consisted of a two-link Plexiglas arm that could be attached to the primate chair and positioned to barely touch the upper arm and evoke abduction. Direct reaches were performed to eight target locations using two different initial postures, one relaxed (default) and one abducted (passively enforced by the apparatus and similar to the anticipatory one recorded in the obstacle block). This experiment was performed after the D1-OA-D2 block sequence was completed. Data from 35 cells were collected in this control experiment.

### Electrophysiology

Recording chambers were placed over the left medial intraparietal area [MIP, (Matelli and Luppino, 2001)] in both animals, contra-lateral to the performing (right) arm. MIP, located within the posterior part of the medial bank of the intraparietal sulcus (IPS), was identified pre-surgically using previously collected magnetic resonance images (Scherberger et al., 2003). Recordings were made at depths ranging from 4–6 mm from the estimated point (by the first encountered background neural activity) of entry into the brain and were therefore within the medial bank of the IPS. Single-neuron activity was recorded extracellularly with tungsten and platinum-iridium microelectrodes (~1–2 MΩ impedance at 1 kHz) mounted on an FHC microdrive (Frederick Haer and Co., Bowdoinham, ME). The raw signal from each electrode was pre-amplified through a headstage, then band-pass filtered and amplified using a time-amplitude window discriminator (Plexon Inc., TX, USA). Neural data and all behavioral events were automatically stored on a computer disc drive for offline analysis.

Activity was sorted using a commercially available on-line spike-sorting application (Sort-Client; Plexon, Inc.). Well-isolated units were identified by their waveform quality and interspike interval (ISI) distributions. Only single-units that had a clearly identified waveform with a signal-to-noise ratio of at least 4:1 were tracked throughout the entire behavioral paradigm. We also confirmed the stability of the waveforms using commercially available software (Wavetracker; Plexon, Inc.). In this study, we focus on the spiking activity of 165 units. For 111 units we saved the waveforms and in these cells the waveforms were reliably held throughout all experimental blocks of a given day. These 111 units were subjected to analyses of waveform shape (described below).

The behavioral procedures to determine the memory response field across experimental conditions (including the control experiment to dissociate posture and temporal-dynamics adjustments in the same neuron) are described in detail previously (Torres and Andersen, 2006). Once a neuron was isolated, its response field was first mapped with the direct center-outreach (D1). If there was a significant directional tuning, recording proceeded to the obstacle-avoidance (OA) block. Here an obstacle was placed close to the cell's preferred location, defined as the location associated with the greatest firing rate over the 800 ms memory period, when averaged over all trials. Two examples of MIP tuning maps during the memory period are shown in Figures 4, 5. In both cases, the neurons had memory activity that was tuned down and to the left of the starting position during D1, thus the obstacle was placed to the left in both cases. At the conclusion of block OA, the obstacle was removed and activity was again recorded as the animal performed the second set of direct reaches (D2).

#### Data analysis

***Kinematic analysis: endpoint (hand) trajectories.*** The kinematic data set under consideration comprises 21 experimental sessions from the two animals. We defined the beginning and the end of movement using 5% of the maximum tangential velocity along the path as a threshold. Data points occurring at time points beyond where the velocity dropped to 5% of maximum were discarded. Various geometric parameters of the corresponding handpaths were quantified including overall path length and path curvature (Torres and Andersen, 2006). An index quantifying the path curvature (K) was calculated by determining the normalized distance from each point along the curved path to its corresponding projection onto the straight line from the starting position to the target position, properly normalized by the largest amount of bending across the data set. According to this method a perfectly straight path would have *K* = 0 bending. The Wilks's lambda test statistic (Rencher, 1995) was used in a standard multivariate ANOVA to examine differences in kinematic parameters between blocks or portions of blocks. For these analyses, only targets in the columns beyond the one where the obstacle was placed (i.e., those where the kinematics could potentially be affected by the obstacle) were analyzed. Typically, this was all three targets in the column.

***Kinematic analysis: postural trajectories.*** Posture paths for seven joint angles were obtained from the Polhemus sensors placed on the shoulder [three rotational joints anchored at the shoulder representing abduction-adduction, flexion-extension and pronation-supination (i.e., rotation about the humeral axis)], elbow (two rotational joints anchored at the elbow representing flexion-extension and pronation-supination), and wrist [two rotational joints anchored at the wrist representing flexion-extension and abduction-adduction, see details in (Torres and Zipser, 2002; Torres et al., 2011)]. Prior to statistical analyses, each path in posture space was resampled to have the same number of points (100) without altering the shape of the curve. Analysis of the arm postural parameters focused on movements to the six target locations (three on the right ends of the board and three on the left ends of the board, depending on the obstacle's location) in the vicinity of the obstacle (Table 1; T1–T6). Comparisons included: (1) D1 vs. OA1; (2) D1 vs. OA2; (3) OA1 vs. OA2; and (4) D1 vs. ABD (from the control experiment). The tested hypotheses included: (1) whether or not the postural paths were the same and (2) whether or not the initial and the final postures remained the same across trials. The Wilk's lambda test statistic (Rencher, 1995) was obtained for each point along the mean postural paths and the average lambda over the 100 points 21 sessions was calculated. In Table 1, the posture path entries represent the mean postural path lambda value across the 21 sessions ± the standard deviation from the mean. The individual averaged lambda values for the initial and final postures are also shown.

**Table 1:** **Results of statistical analyses of postural and endpoint trajectories**.

	**T1**	**T2**	**T3**	**T4**	**T5**	**T6**
**D1 vs. OB1**						
Posture path	0.007 ± 0.003	0.004 ± 0.002	0.01 ± 0.005	0.001 ± 0.05	0.006 ± 0.005	0.005 ± 0.001
Initial posture	0.012	0.006	0.00002	0.007	0.003	0.003
Final posture	0.005	0.011	0.008	0.002	0.002	0.004
**D1 vs. OB2**						
Posture path	0.005 ± 0.001	0.005 ± 0.003	0.02 ± 0.003	0.001 ± 0.05	0.004 ± 0.001	0.006 ± 0.001
Initial posture	0.015	0.004	0.0003	0.005	0.001	0.003
Final posture	0.003	0.011	0.0001	0.005	0.002	0.005
**OB1 vs. OB2**						
Posture path	0.37 ± 0.02	0.35 ± 0.05	0.29 ± 0.03	0.39 ± 0.05	0.31 ± 0.05	0.29 ± 0.07
Initial posture	0.21	0.34	0.26	0.35	0.30	0.31
Final posture	0.37	0.32	0.28	0.37	0.32	0.27
**D1 vs. ABD INIT POSTURE**						
Posture path	0.002 ± 0.001	0.001 ± 0.005	0.006 ± 0.001	0.005 ± 0.001	0.003 ± 0.002	0.002 ± 0.001
Initial posture	0.002	0.005	0.001	0.001	0.001	2 × 10^−4^
Final posture	0.002	0.003	0.001	0.003	1 × 10^−4^	0.0007
**HAND PARAMETERS**						
		**D1 vs. OB1**				
δ distance to peak velocity (different)	*H* = 1,	*H* = 1,	*H* = 1,	*H* = 1,	*H* = 1,	*H* = 1,
	*P* = 0	*P* = 0.0001	*P* = 0.007	*P* = 0	*P* = 0	*P* = 0.0005
Path length (different)	*H* = 1,	*H* = 1,	*H* = 1,	*H* = 1,	*H* = 1,	*H* = 1,
	*P* = 0.004	*P* = 0.003	*P* = 0.0002	*P* = 0.0007	*P* = 0.0009	*P* = 0.005
τ time to peak velocity (different)	*H* = 1,	*H* = 1,	*H* = 1,	*H* = 1,	*H* = 1,	*H* = 1,
	*P* = 0	*P* = 0.0005	*P* = 0.0001	*P* = 0	*P* = 0	*P* = 0.0001
		**D1 vs. OB2**				
δ (different)	*H* = 1,	*H* = 1,	*H* = 1,	*H* = 1,	*H* = 1,	*H* = 1,
	*P* = 0	*P* = 0.001	*P* = 0.005	*P* = 0	*P* = 0.0002	*P* = 0
Path length (same)	*H* = 0,	*H* = 0,	*H* = 0,	*H* = 0,	*H* = 0,	*H* = 0,
	*P* = 0.68	*P* = 0.42	*P* = 0.45	*P* = 0.78	*P* = 0.65	*P* = 0.83
τ (same)	*H* = 0,	*H* = 0,	*H* = 0,	*H* = 0,	*H* = 0,	*H* = 0,
	*P* = 0.83	*P* = 0.98	*P* = 0.93	*P* = 0.67	*P* = 0.98	*P* = 0.57
		**OB1 vs. OB2**				
δ (different)	*H* = 1,	*H* = 1,	*H* = 1,	*H* = 1,	*H* = 0,	*H* = 1,
	*P* = 0	*P* = 0	*P* = 0	*P* = 0	*P* = 0.9	*P* = 0.002
Path length (different)	*H* = 1,	*H* = 1,	*H* = 1,	*H* = 1,	*H* = 0,	*H* = 1,
	*P* = 0	*P* = 0	*P* = 0	*P* = 0	*P* = 0.6	*P* = 0
τ (different)	*H* = 1,	*H* = 0,	*H* = 1,	*H* = 1,	*H* = 1,	*H* = 0,
	*P* = 0.012	*P* = 0.53	*P* = 0.02	*P* = 0	*P* = 0.001	*P* = 0.67

**Figure 2:**
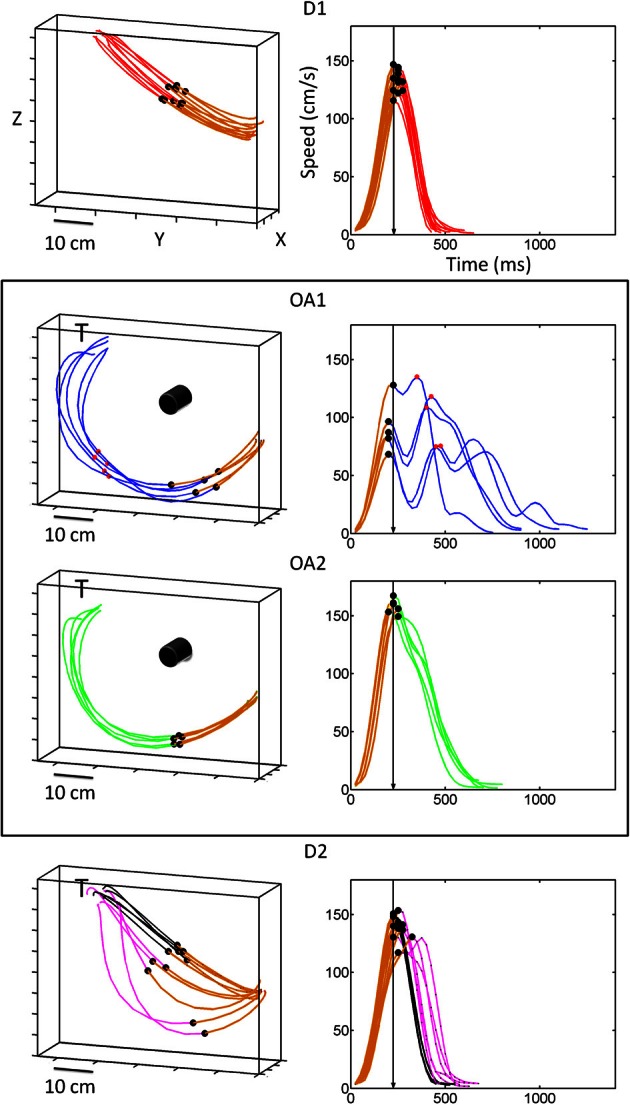
**Hand kinematics for movements to a single target, with and without an obstacle present.** In the absence of an obstacle, handpaths (left panels) were straight and velocity profiles (right panels) were single peaked. The dots along the trajectory mark the first velocity peak of the trial. The first segment colored in brown marks the distance traveled up to the first velocity peak. This brown segment in the hand path corresponds to the acceleration phase of the reach, the initial portion of the speed profile, and the black dot on the speed profile is the first velocity peak also marked along the hand path. The other dots on the speed profile mark additional peaks along the path. The arrow in the speed profile marks the time to the first peak, which was the same across each target location despite differences in the distance traveled up to the first peak. The value of the first peak was adjusted by gradually varying the distance at a constant time. During OA1 and OA2, hand paths to this target became curved but were consistently smooth across trials and joints. In contrast, timing (in the deceleration phase) was highly variable in OA1 and only became consistent during OA2. During the first few trials of D2, an aftereffect of the obstacle was initially observed (pink traces), but movements rapidly reverted to the pattern of kinematics exhibited in D1.

Statistical analysis of the arm postural and hand spatial parameters during the learning of the arm motion dynamics in the trajectories to all six most affected target locations (T1–T6) marked in Figure 2 in the main text. Comparisons include: (1) straight reaches (straight) with the early phase of obstacle-avoidance where the learning of new temporal dynamics took place; (2) early obstacle-avoidance (speed learning) with late obstacle-avoidance (no speed learning); (3) straight initiated from a normal (relaxed) arm posture vs. straight initiated from a (passively enforced) adducted posture. Next to the numbers (1), (2), and (3) we state the statistically chosen hypothesis (same or different) for the corresponding parameter.

At the extrinsic (hand) level we asked whether or not the total path length (cm) to the target, the partial distance traveled up to the maximum velocity (δ cm) and the time to reach that distance (τ ms) were the same or different. A two-tail t-test at the alpha level of 0.01 was performed on the δ, the path length and τ for each affected target. The Matlab convention is H = 1 rejects the null hypothesis that the means are equal at the 0.01 significance level and H = 0 otherwise. Each entry has the P-value as well.

At the intrinsic (arm postural) level, the tested hypotheses included: (1) whether or not the postural paths were similar (remained invariant to speed learning); (2) whether or not the initial and the final postures remained the same across trials (i.e., did not relaxed toward the normal default posture to initiate straight reaches).

Postural analysis (Table 1) for seven joint angles of the arm was obtained using the Wilk's lambda (Rencher, 1995). Each path in posture space was resampled to have the same number of points (100) without altering the shape of the curve. Each target was treated independently with k number of conditions (e.g., k = 2 for learning vs. automatic). The Wilk's lambda test was performed on each separate point (seven-dimensional vector) along the path for the postural paths of five trials.

Wilk's lambda statistic has the likelihood ratio test $\Lambda =\frac{det(E)}{det(E+H)}$ written in terms of the “within” sum of squares and products matrix E and the “total” sum of squares and products matrix (E + H). The matrix $E=\sum_{ij}y_{ij}y_{ij}^{t}-\sum_{i}^{k}\frac{1}{n}y_{i.}y_{i.}^{t}$ where y_ij_ is a sample point and $y_{i.}=\sum_{j}^{n}y_{ij}$ is the total sum of the i-th sample. The matrix $H=\sum_{}^{k}\frac{1}{n}y_{i.}y_{i.}^{t}-\frac{1}{kn}y_{..}y_{..}^{t}$ where $y_{..}=\sum_{i}^{k}\sum_{j}^{n}y_{ij}$ is the overall total. This test is similar to the univariate F-test. The use of determinants reduces the test statistic Λ to a scalar, making it possible to decide whether the separation of mean vectors is significant. When Λ ≤ Λ _α, d, νH,νE_ (Λ small, the null hypothesis is rejected. In Λ_α, p, ν H,νE_, α is the level of confidence, d is the number of variables or dimension, νH = k − 1 and νE = k(n − 1) are the degrees of freedom for hypothesis and error, respectively.

The Wilk's lambda rule rejects the null hypothesis of mean equality for Λ ≤ Λ^*^_α, d, νH, νE_ where α = 0.05, d = 7, and ν_H_ = 2 − 1, ν_E_ = 2(5 − 1), are the degrees of freedom for hypothesis and error terms, respectively for the joint-angle paths. The number of samples k = 2, (straight vs. obstacle-learning, obstacle learning vs. automatic, and straight normal vs. abducted initial posture). Each block has 10 trials, which were divided into 5 early and 5 late. Thus the number of points per sample-condition is n = 5. Λ^*^_α = 0.05, d = 7, ν_H_ = 1, ν_E_ = 8_= *0.176* taken from Rencher, 1995).

The data set under consideration comprises 21 experimental sessions from the two animals. These were within the experimental days where each day we tested the same cell in the initial-abducted-posture control experiment with their corresponding blocks of obstacle-avoidance.

Joint angle paths were obtained from the Polhemus sensors (Fastrack System 120 Hz sampling resolution). Positional sensor paths were re-sampled to have 100 points and postural paths for 7 joint angles were obtained. These postural arm paths reconstructed the sensor positional paths. Lambda values were obtained for each point in each of the postural paths and for each set the lambda value for a session-sample was taken as the average lambda over the 100 lambda-points of the postural paths. Entries are the mean postural-path lambda value across the 21-session samples ± the standard deviation from the mean. The individual averaged lambda values for the initial and final postures are also shown.

***Statistical analysis of neural data.*** Two-Way ANOVAs were used to assess the dependence of mean firing rate on target location and the various conditions (i.e., blocks or portions of blocks). Previous analyses had demonstrated that handpaths and velocity profiles in this task differed statistically for some targets between the initial block of direct reaches (D1) and the first five trials of block OA (Torres and Andersen, 2006). In addition, although handpaths were statistically indistinguishable between the first five trials and the last five trials of OA, temporal profiles were generally statistically different. As a result, separate Two-Way ANOVAs were performed comparing activity in D1 to the first five trials of OA (“OA1”) and OA1 to the last five trials of OA (“OA2”). A comparison between D1 and OA2 was also performed to assess if differences persisted after the speed profiles along the curved trajectory had turned unimodal. In addition, following removal of the obstacle, i.e., during D2, after-effects were often observed in the trajectories during the first few trials, after which the animal “de-adapted” to the presence of the obstacle. Thus, a separate ANOVA was also performed comparing the activity in D1 to the last five trials of D2 to determine whether or not activity returned to its pre-obstacle state.

***Alignment analysis of response fields.*** To further quantify differences in individual cell responses between blocks we used methods derived from differential geometry. Specifically, we first formed matrices of the scalar mean firing rates for each target location. We used interpolation techniques using the Spline toolbox from MATLAB to obtain a less coarse representation of the board. We then converted the matrices into vector field representations and computed the Lie Bracket, which is equivalent to computing the derivative of one vector field with respect to another (Carmo, 1976; Gray, 1998). The resulting residual field is another vector field that indicates the magnitude and direction of change in the fields along each dimension. In cases where the original vector fields are very similar the residual field vectors will be of negligible magnitude throughout. In this experiment, where fields are compared between blocks with and without the obstacle, a residual field of negligible magnitude would mean that the two tuning fields are *aligned*, implying invariance with respect to the changes evoked by the obstacle-avoidance task. A neuron exhibiting these types of responses could be interpreted as a task-relevant cell whose rates are tuned to the target location/movement vector in endpoint space, but whose responses are invariant with respect to differences in trajectory. In cases where the original vector fields are different, the residual fields will not be negligible in magnitude and can provide insight into whether the original fields are rotated or rotated-and-scaled versions of each other. In such cases the two tuning fields derived from the same neuron are *misaligned* and provide information regarding the trajectory planning dependencies of that cell.

***Analysis of spike waveform.*** Previous studies have identified putative interneurons and pyramidal neurons in sensory and motor areas of the cortex on the basis of waveform shape along with evidence for distinct contributions of these populations to neural coding (Mitchell et al., 2007; Chen et al., 2008; Kaufman et al., 2010; Song and McPeek, 2010). As a result, we looked for evidence of distinct neuronal populations here as well. To study the distribution of the spike widths we used the waveforms from all trials and all experimental blocks. Only well-isolated units, identified according to the criteria described above, were analyzed further. Action potentials from each cell were first aligned by their troughs and averaged. The resulting waveforms were typically biphasic; neurons exhibiting waveforms that were not biphasic with a clear peak were excluded from further analysis. Waveforms from the remaining neurons (111 altogether) were amplitude-normalized and their trough to peak durations (in μs) were calculated. The resulting distribution of spike widths was then subjected to Hartigan's dip test of unimodality (Hartigan and Hartigan, 1985).

#### Decoding analysis

To assess how well MIP population activity could predict movement trajectories under different conditions we used nearest-neighbor decoding techniques (Quian Quiroga and Panzeri, 2009). For these analyses, cells were considered to be simultaneously recorded. For each cell and trial, the mean firing rate during the memory period was used as input to the decoding algorithm to predict various aspects of the impending behavior in a given block of trials. Five trials of data per target location were used in any given condition. For the blocks of direct reaches in both the main and control experiment data from all target locations were used. During block OA, the obstacle typically prevented movements to 1–2 locations. As a result, only the five board locations that were common to all cells were analyzed in this block.

Trials were represented as points in an *m*-dimensional space, each coordinate corresponding to the mean firing rate over the 800 ms memory period for each of the *m* cells. One at a time, data from each trial was used to predict the target/trajectory, based on distributions derived from all remaining trials (leave-one-out cross validation) and was assigned to the class of its nearest neighbor in the *m*-dimensional space using Euclidean distance (Duda et al., 2001). A complete description of the method, as well as corresponding graphical depiction can be found in (Quian Quiroga et al., 2006; Quian Quiroga and Panzeri, 2009). For assessing statistical significance of the decoding results, a value of 1 was assigned to correctly predicted trials and a value of 0 to the incorrectly predicted ones. The mean of the sequences of correctly and incorrectly classified trials were compared statistically using a non-parametric Wilcoxon rank-sum test (Zar, 1996) and were represented graphically as confusion matrices.

## Results

### Behavioral results

Hand trajectories were altered substantially once animals learned to avoid the obstacle, as compared to direct (straight) reaches to the same target, and these spatial and temporal alterations were partially dissociated in time (Figure 2). The spatial paths that successfully avoided the obstacle were resolved immediately and remained consistent in three dimensions. On very few trials the hand collided with the obstacles, suggesting that in complete darkness the animals immediately solved the proper curvature of the avoidance path. In contrast, the temporal aspects of the trajectories took longer to master. More specifically, we observed a gradual learning process in which both animals transitioned from smooth multi-peaked velocity profiles along the hand path (OA1) to smooth unimodal velocity profiles (OA2). This learning occurred at two main time scales that we tracked longitudinally across months, one short-term (daily) and one long-term (across months). On the daily time scale, learning of appropriate trajectories was associated with a de-adaptation process that was observable and quantified as “after-effects” following removal of the obstacles (see below). Eventually, upon months of training these after-effects gradually disappeared and the transition from OA to D2 was more direct than in the initial days and weeks of exposure to the obstacle. A full description of these kinematic changes has been reported previously (Torres and Andersen, 2006; Torres, 2010) and are only briefly summarized here.

Figure 2 shows example handpaths and speed profiles for movements to the target at the upper leftmost position during the first block of direct reaches (D1), the early trials of obstacle avoidance (OA1), the later trials of obstacle avoidance (OA2) and the last block of direct reaches when the obstacle was removed upon adaptation to the obstacle (D2) (top to bottom). In D1, handpaths were relatively straight and velocity profiles were bell-shaped, with peak velocity (black dots) occurring at a consistent time point. During the first five trials of the obstacle avoidance block (OA1), animals rapidly adopted curved but consistent handpaths that would allow them to successfully avoid the obstacle. Importantly, however, the velocity profiles during these initial trials were *not* consistent and were typically multi-peaked. This behavior changed gradually during the obstacle block such that by OA2 animals returned to the single-peaked velocity profiles exhibited in D1. After the removal of the obstacle (D2), a “de-adaptation” was observed where the first few movements were still noticeably curved despite the fact that the obstacle was no longer present (pink traces). This was true even though the animals observed the obstacle being removed from the board between blocks and were therefore aware of the change in task conditions. In the second half of D2, the kinematics recovered and approximated those seen in D1 (black traces).

These observed similarities and differences in endpoint kinematics between blocks were confirmed statistically on a daily basis and across months as a global effect across the board (Torres and Andersen, 2006; Torres, 2010). These analyses showed that several aspects of the handpaths, including the overall path length and curvature, differed significantly between D1 and OA1 for all examined targets (Wilks test statistic; *p* < 0.01, Table 1) and for many targets the number of velocity peaks also differed (*p* < 0.01). Although handpaths were statistically indistinguishable within the obstacle block (i.e., between OA1 and OA2), movements to some targets were associated with differing numbers of velocity peaks, indicating highly variable temporal dynamics. Regarding the effects of obstacle removal, most features of the kinematics differed significantly between OA2 and the first half (five trials) of D2 and between the first half and last half (five trials) of D2, consistent with the process of de-adaptation described above. Lastly, when trajectories during the last five trials of D2 were compared with those in D1, virtually all kinematic parameters were statistically indistinguishable, indicating that animals exhibited nearly full de-adaptation to the presence of the obstacle by the end of D2.

### Neurophysiology: effect of obstacle

**Figure 3:**
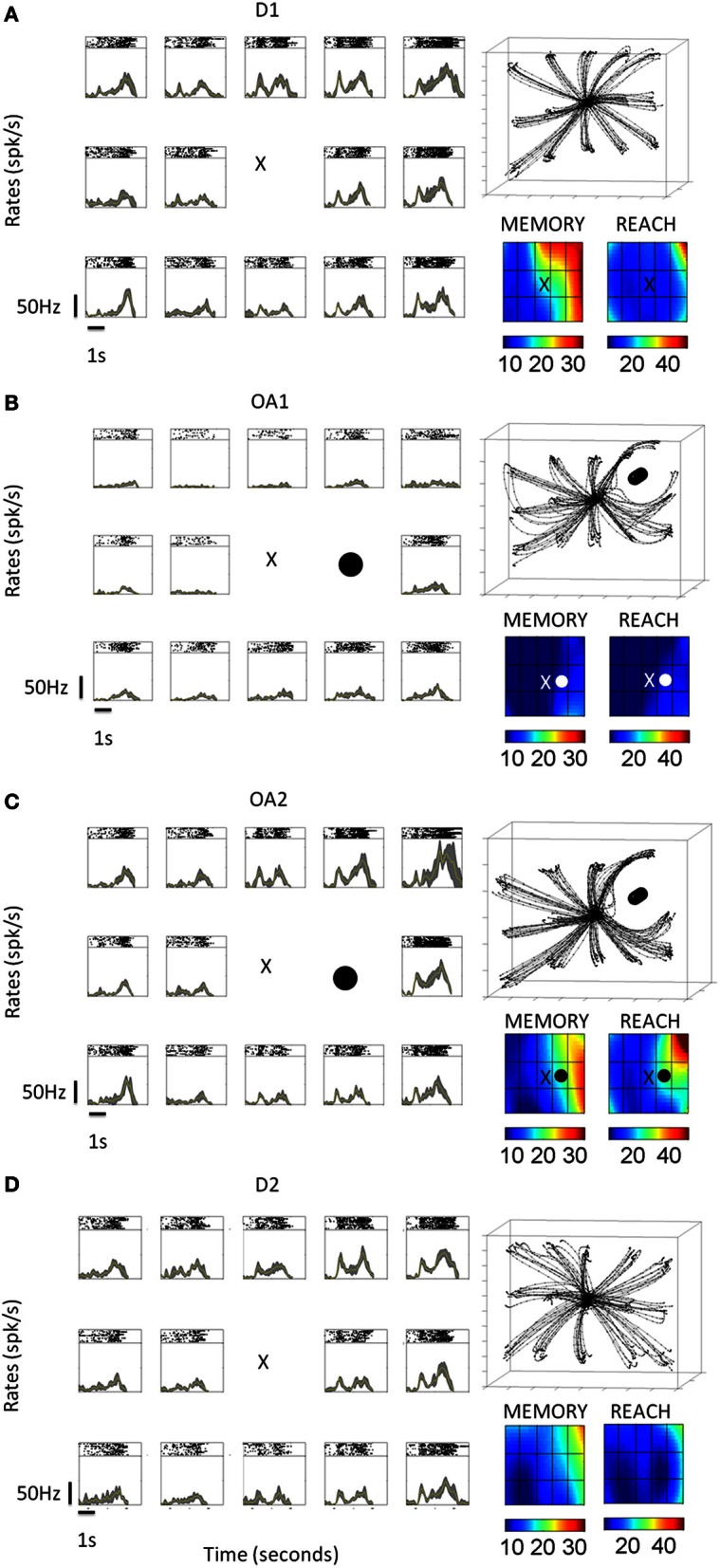
**Neural activity from a single example cell under different conditions (D1, OA1, OA2, and D1, shown top–down as time progress).** The concurrently recorded hand trajectories are also shown for each block. **(A)** D1 activity across all epochs (baseline, cue, memory, and reach) for 14 board locations. Color maps built by interpolating the mean firing rates across board locations during the memory and reach epochs are shown at the lower right. **(B)** Activity and behavior during the first five trials of the OA block (OA1). Black circle indicates the board location blocked by the physical obstacle. **(C)** Activity and behavior during the last five trials of the OA block (OA2). **(D)** Activity and behavior during the 2nd block of direct reaches (D2).

The activity of many MIP neurons was suppressed during the initial phase of the obstacle avoidance block, across all epochs compared to the initial block of direct reaches. Figure 2 shows the changes in kinematics during the early trials of the obstacle avoidance block. The planning activity preceding these motions changed and recovered in tandem with changes in several kinematic parameters until the motions became consistent. Notably, while the hand paths remained stable, the animals performed exploratory rather than ballistic movements characterized by intermittent “pauses” that changed the tempo of the reach from trial to trial until the speed stabilized. Detailed kinematic analyses revealed that the speed profiles changed gradually with differences between the acceleration and the deceleration phase of the trajectory. During the acceleration phase, across all targets the time to the peak velocity remained stable as the distance traveled by the hand gradually changed. In contrast the deceleration phase had variable timing which gradually decreased as the speed turned faster and the tempo acquired consistency. Figure 3 shows PSTHs of the activity (left columns) during the entire task, as well as concurrently recorded handpaths and color maps of the mean firing rates during the memory and reach epochs for a single example cell As illustrated in Figures 2, 3A shows that in block D1, the animal moved on approximately straight paths to the targets and cell activity was tuned to the upper right portion of the target array. Immediately following the placement of an obstacle to the immediate right of the starting position (OA1), the animal performed dramatically different movements to the remaining targets on the right. Movements to the other targets, though similar to those in D1, were more variable. This increased variability was also reflected in the velocity profiles (not shown here but see Figure 2). Regarding the cell activity, firing rates were dramatically reduced for virtually all target locations and the tuning also changed such that activity was greatest for locations to the lower rather than upper right. In the latter half of the obstacle avoidance block (OA2), the overall activity as well as the spatial tuning of the cell recovered close to that in D1, particularly during the reach epoch. This recovery was associated with more reliable handpaths and velocity profiles, even though the paths to the right targets were still very different from those in D1. Lastly, after the obstacle was removed (D2), the spatial and temporal aspects of the kinematics, as well as the cell activity, very closely approximated those in D1.

**Figure 4:**
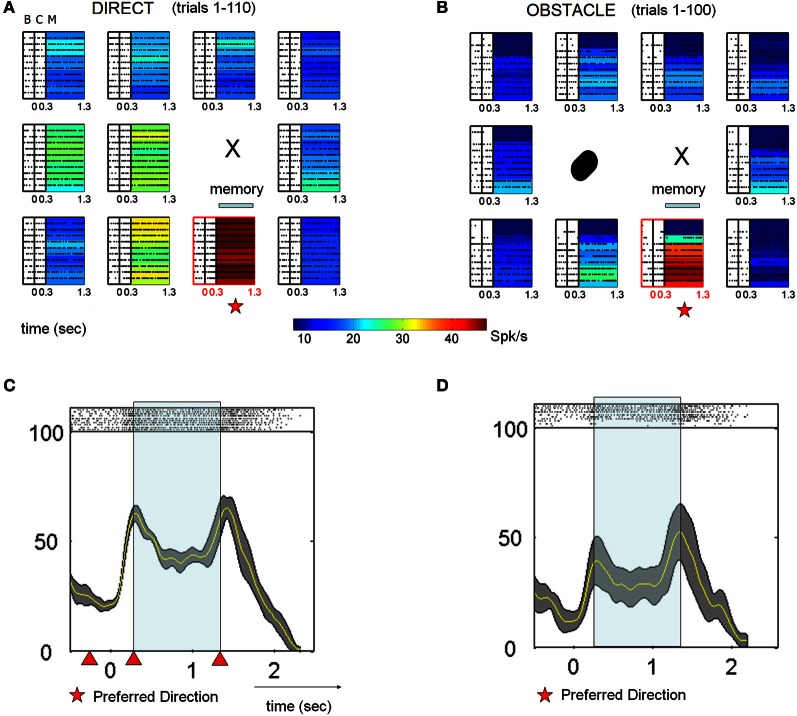
**Typical activity of single cell suppressed in the presence of an obstacle across all epochs. (A)** For these spike rasters, earlier trials are the bottom rows. The letters denote the epochs (baseline B 300 ms, cue C 300 ms, memory M 800–1000 ms). Pre-movement activity during direct reaches for the baseline, cue and memory epochs. In the absence of an obstacle the neuron was tuned down and to the left, with the preferred location in the memory period directly below the starting position (X). Color-coded maps represent the mean firing rates at each location. **(B)** Activity in the presence of the physical obstacle placed on the left. Activity was strongly suppressed during the first few trials then gradually recovered. The preferred location of the neuron remained the same during this block. **(C,D)** Peristimulus time histograms (PSTHs) of spike activity, along with spike rasters are shown for the preferred location. Experimental blocks depicted are D1 and OA (early learning and late skilled trials). Triangles mark the ending of the baseline, cue and memory in that order from left to right.

Figure 4 shows the activity of another MIP neuron for reaches to 11 board locations during block D1 **(A)** and block OA **(B)** in a different format. This cell was tuned down and to the left of the starting position during the memory period of D1. Consequently, the obstacle was placed to the left of the starting position. During the first few trials of OA (top lines of rasters in panel B, bottom lines of rasters in panel D) the activity of this neuron was markedly suppressed. For some locations the cell appeared not to fire at all for the first few trials. As the block progressed, activity began to gradually recover, with the rate of recovery differing somewhat for the different board locations. By the end of the OA block, however, the firing rates of this cell, as well as the rates of most other cells (detailed in Table 2) exhibiting response suppression, did not return completely to those observed during D1. The preferred location of the neuron based on the memory activity (stars) remained the same throughout both blocks.

**Table 2:** **Results of 2 factor ANOVA of memory period activity for all cells**.

		**Location (%)**	**Condition (%)**	**Interaction (%)**
D1 vs. OA1	Enhanced	35/43 (81.4)	27/43 (62.8)	9/43 (20.9)
	Suppressed	40/68 (58.8)	45/68 (66.2)	6/68 (8.82)
D1 vs. OA2	Enhanced	31/43 (72.1)	30/43 (68.8)	5/43 (11.6)
	Suppressed	42/68 (61.8)	48/68 (70.6)	10/68 (14.7)
OA1 vs. OA2	Enhanced	38/43 (88.4)	17/43 (39.5)	2/43 (4.6)
	Suppressed	46/68 (67.6)	32/68 (47.0)	2/68 (3.2)
D1 vs. D2	Enhanced	36/43 (83.7)	21/43 (48.8)	6/43 (13.9)
	Suppressed	46/68 (67.6)	38/68 (55.8)	6/68 (8.82)

Pairwise comparison across two consecutive blocks of the changes in firing rates during the memory epoch to assess significance levels using Two-Way ANOVA with location and condition as the factors, with alpha 0.01. These analyses included 111 neurons for which the waveforms were saved (out of 165 neurons).

**Figure 5:**
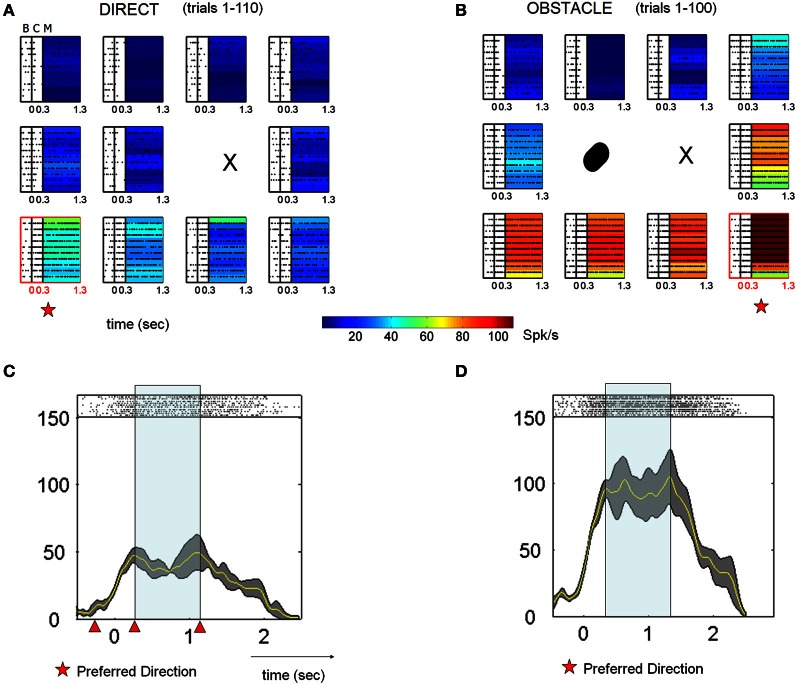
**Single cell response enhancement in the presence of an obstacle. (A)** Data from 10 consecutive trials (top to bottom) aligned to cue onset are shown, with the raster arrays color coded according to the mean firing rate during the memory period. (All symbols as in Figure 4). **(B)** This neuron demonstrated an immediate increase in firing rate when moving in the presence of the obstacle. From D1 to OA, the preferred location transiently differed between conditions. Note that the neuron began to reduce its firing rate over the last few trials for most locations. **(C,D)** Peristimulus time histograms (PSTHs) of activity associated with the preferred location, along with spike rasters. Earlier trials are the bottom rows and triangles mark the ending of the baseline, cue and memory in that order from left to right.

The activity of other neurons was strongly enhanced in the presence of the obstacle. Figure 5 shows the response of one neuron in the same format as Figure 4. This neuron was tuned to the lower board locations during block D1, with a slight preference for the lower leftmost location. In the presence of the obstacle, activity increased dramatically for these lower board locations as well as for the location immediately to the right of the starting position. This increase was maintained throughout most of the obstacle avoidance block, with noticeable decreases coming only very late in the block and with the rate of recovery again differing for the different board locations. In contrast to the previous cell shown in Figure 4, the preferred location of this neuron shifted between blocks. Although transient and statistically significant shifts in the preferred location during OA were observed for other neurons as well, by the end of D2 the preferred locations of most cells returned to those exhibited in D1. The number of cells and percentages are detailed in Tables 2, 3.

**Table 3:** **Results of 2 factor ANOVA of reach period activity for all cells**.

		**Location (%)**	**Condition (%)**	**Interaction (%)**
D1 vs. OA1	Enhanced	23/43 (53.5)	28/43 (65.1)	2/43 (4.7)
	Suppressed	30/68 (44.1)	51/68 (75.0)	5/68 (7.4)
D1 vs. OA2	Enhanced	24/43 (55.8)	26/43 (53.5)	1/43 (2.3)
	Suppressed	32/68 (47.1)	48/68 (70.6)	13/68 (19.1)
OA1 vs. OA2	Enhanced	22/43 (51.1)	19/43 (44.2)	3/43 (6.9)
	Suppressed	35/68 (51.5)	30/68 (62.5)	3/68 (4.41)
D1 vs. D2	Enhanced	16/43 (37.2)	33/43 (76.7)	5/43 (11.6)
	Suppressed	29/68 (42.6)	37/68 (54.4)	2/68 (2.9)

Pairwise comparison across two consecutive blocks of the changes in firing rates during the reach epoch to assess significance levels using Two-Way ANOVA with location and condition as the factors, with alpha 0.01. These analyses included 111 neurons for which the waveforms were saved (out of 165 neurons).

Cells exhibiting statistically significant suppression (110/165) and enhancement (55/165) were roughly balanced across the population, similar to the approximately balanced increases and decreases in M1 and PMd activity during visuomotor adaptation (Wise et al., 1998). Statistically significant changes in firing rate between and within blocks were assessed in each cell using a Two-Way ANOVA [factors: location and condition (block/portion of block)]. Cells were first assigned by a computer program to either a suppression or enhancement group, based on the differences of rate changes computed between D1 and OA1, then subjected to statistical analysis. If the difference (OA-D1) in the mean firing rate of the cell during the memory period was negative, the cell was assigned to the suppression group. It the difference was positive, the cell was assigned to the enhanced group. When responses in D1 were compared to those in OA1, most cells demonstrated statistically significant main effects of location and condition, though few cells demonstrated interaction effects (Table 2). This paucity of interaction effects suggests that initial enhancement and suppression were global phenomena affecting all examined locations in a similar way. The proportion of cells showing a main effect of location was similar for the other comparisons as well. Regarding changes observed between OA1 and OA2, a substantial number of neurons showed an effect of condition, meaning that activity was significantly different during the memory period preceding the movement for impending movements with similar spatial characteristics but different speed profiles. A similar proportion of neurons showed an effect of condition when D1 vs. D2 were compared (Table 2). This is related to the fact that de-adaptation to the presence of the obstacle was slow and variable across neurons and also involved changes in the postural paths associated with movements to particular target locations. We touch on this point again in following two sections.

### Suppression vs. enhancement: relation to spike width

**Figure 6:**
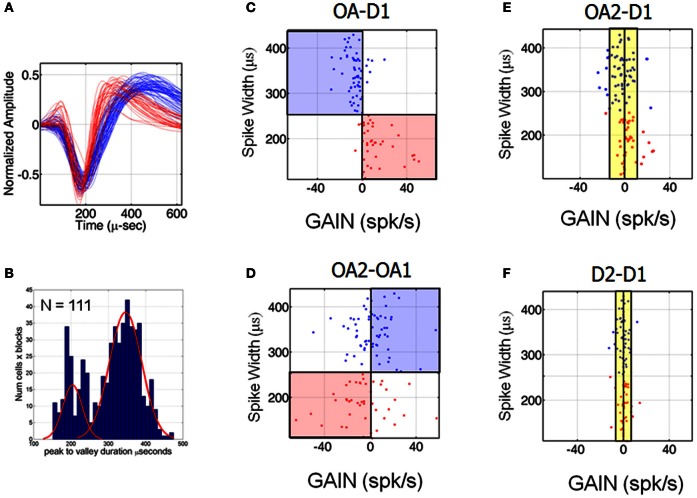
**Cells classes based on different spike widths were associated with different patterns of suppression and enhancement. (A)** Average waveforms of neurons belonging to the two groups indicated by the mixture of Gaussian fit illustrated in **(B)**. **(B)** Histogram of spike widths for all trials, blocks and neurons. **(C–F)** Scatter plots of spike width vs. change in firing rate (at the preferred location) for all neurons. The change (denoted here GAIN) was positive if OA-D1 difference in firing rates at the preferred location >0 and negative GAIN if <0 (no cells manifested 0 GAIN). The evolution of the changes associated with transitioning from D1 to OA1 **(C)**, OA1 to OA2 **(D)**, D1 to OA2 **(E)** and from D1 to D2 **(F)** are shown.

Several recent neurophysiological investigations have identified populations of putative pyramidal neurons and interneurons in the visual and premotor cortices, based on the relative widths of their extracellularly recorded spikes (Csicsvari et al., 1998; Mitchell et al., 2007; Chen et al., 2008; Kaufman et al., 2010; Song and McPeek, 2010). As a result, spike waveforms recorded here were examined offline to assess the distribution of spike widths in the population. Figure 6A shows a plot of two groups of waveforms to illustrate that waveforms segregated analytically by spike width (as described in Materials and Methods and below) were clearly distinguishable. Figure 6B shows a histogram of the time differences between maximum and minimum voltage deflections for all trials, blocks and neurons for which spike waveforms were saved. This distribution was significantly non-unimodal (Hartigan's dip test, *p* < 0.001) (Hartigan and Hartigan, 1985) and further examination of the histogram suggested the presence of two distributions, with a spike width cutoff of ~250 μs (according to a mixture of Gaussians fitting). We therefore split the neurons into two groups based on this cutoff value and examined the average waveforms of neurons belonging to each group, which turned out to form two classes (as illustrated in Figure 6A). Thus, our analysis of waveform widths suggested the existence of at least two distinct groups of neurons in this population, one relatively narrow spiking and the other relatively broad spiking, as described in recent investigations of other cortical areas.

Interestingly, neurons with different spike widths were associated with different patterns of response suppression and enhancement across blocks. Figures 6C–F show scatter plots of spike width vs. change in firing rate at the preferred location for all neurons, abbreviated by the word GAIN, which could be positive or negative. Positive GAIN refers to an increase and negative GAIN refers to a decrease in mean firing rates at the preferred location of the neuron during D1. Recall here that OA1 refers to the first half of the obstacle block and OA2 refers to the second half. Changes in firing rate associated with moving from D1 to OA1 **(C)**, OA1 to OA2 **(D)**, D1 to OA2 **(E)** and from D1 to D2 **(F)** are shown. Figure 6C shows that cells with wide spikes widths (>250 μs) were generally suppressed at the start of OA block while cells with narrow spike widths were enhanced. This trend changed dramatically, however, over the course of the obstacle block, when responses during OA2 were compared to those in OA1 (Figure 6D). First, note that firing rates were still changing quite dramatically for some neurons during the obstacle block. Some neurons continued to change their firing rates in the same manner as before, i.e., they progressively increased or decreased their firing rates from D1 through OA1 and OA2. In contrast, some cells that had demonstrated a decrease in firing rate from D1 to OA1 crossed the 0-change line in Figure 6D, indicating that firing rates in these cells recovered somewhat as the obstacle block progressed. Recall that impending hand paths were statistically indistinguishable between OA1 and OA2 but animals were still making adjustments from trial to trial in the temporal aspects and speed profiles of these movements. The last two panels in Figure 6 show that as these temporal aspects began to more closely resemble those in D1, firing rates also began to return to their initial levels. That is, activity was more similar between OA2 and D1 than between OA1 and D1 (cf. Figures 6E,C). Activity was even more similar between D1 and D2 (Figure 6F), even though many individual neurons still showed statistically significant differences in activity between these blocks. This is due to the fact that the activity of many neurons did not truly approximate baseline values until late in D2, when spatial and temporal aspects of the movements were generally statistically indistinguishable from those in D1.

### Decoding impending hand trajectories

**Figure 7:**
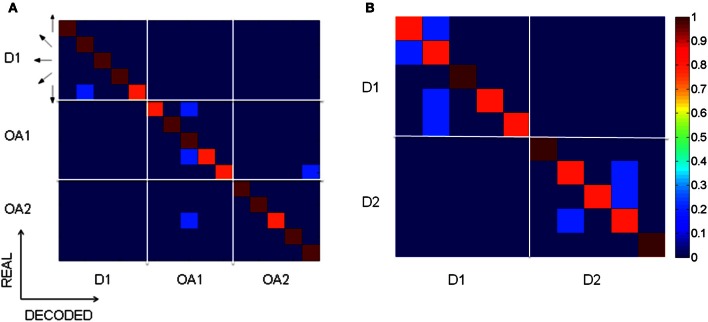
**Decoding analysis of MIP spiking activity within and across blocks.** The confusion matrices show the percentage of trials accurately decoded. Arrows indicate the direction of the target relative to the starting position of the hand. **(A)** The matrices on the main diagonal show the predictions within conditions D1, OA1, and OA2. The off diagonal matrices show that there was no confusion across the different conditions. This means that blocks of trials involving different kinematics were not confused by the decoder, even when these differences were only with regard to temporal dynamics (OA1 vs. OA2). **(B)** Decoding results for conditions D1 and D2.

Decoding analyses showed that at the population level MIP activity distinguished between blocks of trials with different trajectory characteristics. Figure 7A shows the results of the decoding analyses in the form of confusion matrices with actual target location on the ordinate, decoded target location on the abscissa and the proportion of correctly decoded trials indicated by color. Only those target locations that could be compared with and without the obstacle are shown. Using the activity during the memory period, the decoder accurately predicted the upcoming movement direction and did not cause decoding error between different hand kinematics (e.g., D1 vs. OA1). Remarkably, the cells' planning activity also unambiguously distinguished between trials in the early vs. late obstacle block (OA1 vs. OA2), where the hand trajectories differed only with respect to their temporal dynamics. This trend was also observed when decoding directions and conditions in early D2 vs. D1 (Figure 7B). Note that this is not inconsistent with Figure 6F which showed that mean firing rates at the preferred location were generally similar between D1 and D2. The fact that trials in D1 and D2 were not confused by the decoder is a reflection of the fact that decoding is a single trial analysis conducted over the population of neurons and the time course over which neurons recovered after removal of the obstacle was quite variable. That is, although some neurons exhibited a relatively rapid de-adaptation to the obstacle, others de-adapted much more slowly, in parallel with the concurrent trajectory after-effects. The result is also a consequence of anticipatory ***postural changes*** in the memory period, associated with impending movements in the presence of the obstacle and for different targets (see Encoding of Joint Kinematics below), which were often retained for some period of time following obstacle removal. Overall, the decoding analysis suggests that the preparatory activity of MIP population distinguishes among movements involving different endpoint kinematics, even when these movements have similar trajectories but different speed profiles (OA1 vs. OA2).

### Encoding of joint kinematics

**Figure 8:**
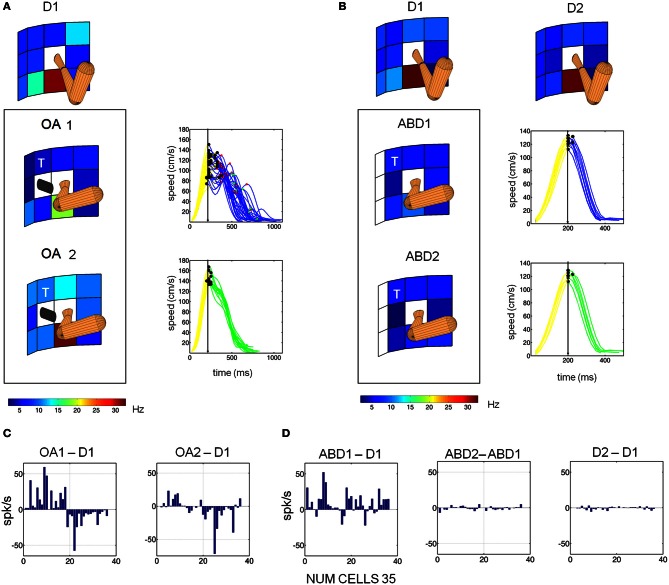
**Voluntary and passive changes in initial arm posture were associated with different changes in single cell activity. (A)** Responses of a single MIP neuron during D1, OA1, and OA2. The activity of this neuron was suppressed during OA1 but partially recovered during OA2 as the velocity profiles became smoother. **(B)** Response of the same neuron during direct reaches (without avoidance) with imposed shoulder abduction (ABD1, ABD2). Activity was suppressed during ABD1 but did not recover in ABD2. Hand velocity also did not evolve. **(C)** Bar plots of the changes in firing rate at the preferred location between OA1 and D1 (left) and OA2 and OA1 (right). Data from 35 neurons are shown. **(D)** Bar plots in the same format as **(C)**, but for the condition involving induced abduction. The “T” marks the board location for which the kinematics are displayed.

As described previously, at the beginning of the obstacle block both animals voluntarily adjusted their initial arm posture (illustrated schematically in Figure 1A). As a result, changes in the memory period activity during the obstacle block could reflect these changes in initial arm posture, modifications in the planned trajectory, or both phenomena. To help distinguish among these possibilities a subset of neurons (35) were examined under three conditions: (1) direct reaches with no obstacle present and a naturally assumed arm posture, (2) reaches in the presence of the obstacle, which involved voluntary changes in initial arm posture (specifically shoulder abduction), and (3) reaches with no obstacle present but with an *experimentally induced* change in shoulder abduction. Figure 8 shows the responses of one neuron tested under these conditions. In the presence of the obstacle, the memory period activity of this neuron was initially suppressed (**8A**: OA1) but recovered as the animal refined the temporal characteristics of its trajectories (OA2). In contrast, when abduction was experimentally induced but no obstacle was present, the memory activity was also suppressed but did not temporally evolve as in the obstacle condition (cf. ABD1 vs. ABD2, Figure 8B). Note also that endpoint kinematics in the ABD condition were statistically indistinguishable from those in D1 and also did not evolve in time (data not shown). Thus, this cell appeared to be affected by both the obstacle-avoidance *and* the change in arm posture, with avoidance having a transient effect and arm posture affecting activity throughout the block. During the final block (D2) the cell recovered closer to D1 than to the OA memory period activity **(8B)**. These findings were representative of the 35 neurons studied under these conditions **(8C,D)**. Transient obstacle avoidance effects were significant in the cells depicted in **8C** (*p* < 0.01). The posture effects were significant in the ABD1-D1 comparisons (*p* < 0.01) shown in Figure 8D. In contrast memory period firing rates were statistically indistinguishable between ABD1 and ABD2 (*p* > 0.8).

**Figure 9:**
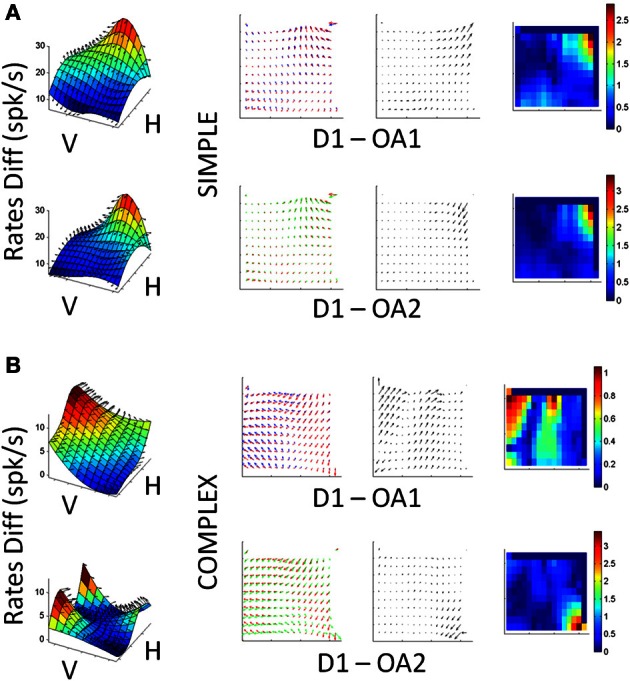
**Single cell responses during changes in arm posture. (A)** Cell showing relatively simple scaling changes with arm posture and speed learning during OA1 and OA2. First column (left to right): Surfaces constructed from the mean firing rate differences between D1 and OA1 (top) and D1 and OA2 (bottom), as a function of board location. Second column: superimposed gradient fields from D1 and OA2, which were used to obtain the Lie Bracket (see Materials and Methods). The resulting residual vector field (third column) is plotted next to a color map of the magnitude of the residual Lie Bracket field (last column). **(B)** Responses of a more complex cell, in the same format as **(A)** where the field rotates and scales with the changes in posture and speed learning from D1 to OA1, then stabilizes in OA2.

We observed both simple and complex changes in cell activity as a result of changes in initial arm posture roughly distributed as 1/3 and 2/3 of the population, respectively. The word “simple” refers to a gain-field effect (such as those quantified using the Lie Bracket in a representative cell in Figure 9A). In such cases the cells maintained their PD across conditions and just manifested a change or modulation in the mean firing rates. In contrast the word “complex” is used to describe the cells that would both change the FR and rotate the PD in complex ways (such as those shown for a representative cell in Figure 9B).

Figure 9A demonstrates a relatively simple scaling of its response field with changes in arm posture. Figure 9B shows complex changes in both the shape and scale of its responses. The surfaces in the first column show the differences in memory period activity between D1 and OA1 (top) and D1 and OA2 (bottom) for all board locations. The 2nd column shows superimposed vector field representations of the mean firing rates during the memory period for each block of trials. The 3rd column shows the residual fields derived from the Lie Bracket analysis (see Materials and Methods); these fields describe how the two vector field representations change with respect to one another in terms of both rotation and/or scaling. The last column shows the change in magnitude only, as a colormap. The cell in Figure 9A exhibited mostly a scaling of its responses in both OA1 and OA2, but the tuning was largely maintained across blocks; this can be appreciated from both the residual fields, which appear relatively laminar, and the colormaps of the changes in magnitude. In contrast, the cell in Figure 9B showed differences in both shape and scale, as evidenced by the more turbulent residual fields, and these changes differed between OA1and OA2. Simple and complex sub-populations included both broad and narrow spiking cells. These observations, when combined with those described in Figure 8, suggest that the observed changes in activity in the presence of the obstacle were due to changes in both the initial postural conditions for avoidance as well as an evolution of arm kinematics over the course of the obstacle block, with separable effects sometimes observable in the same neuron (e.g., Figure 9B).

**Figure 10:**
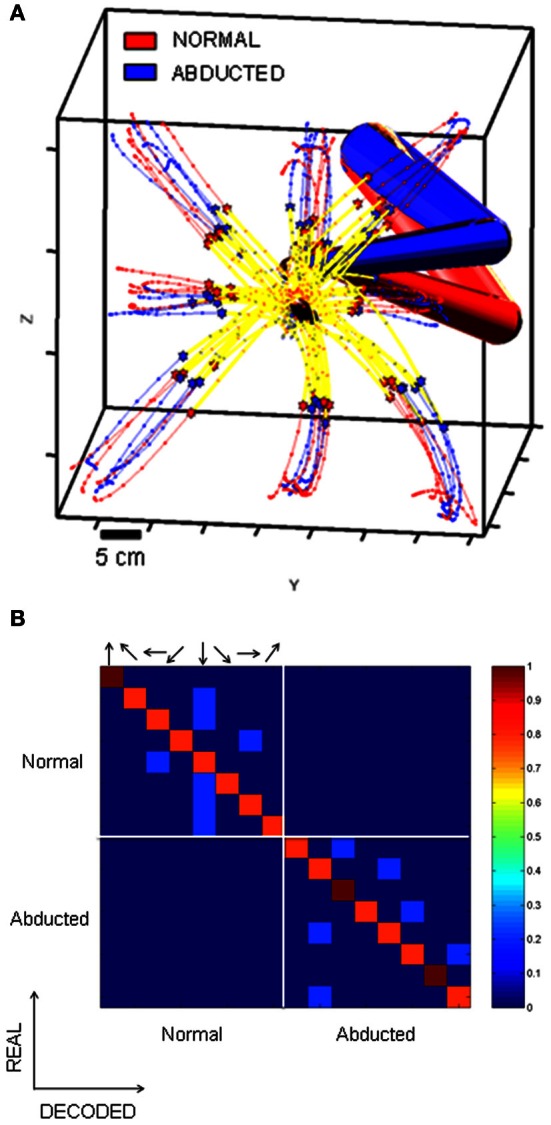
**Decoding analysis of movements with similar endpoint kinematics but different joint kinematics.** The confusion matrices show the percentage of trials accurately decoded. Arrows indicate the direction of the target relative to the starting position of the hand. **(A)** Endpoint kinematics for sets of movements involving different initial arm postures and therefore different joint kinematics. Endpoint kinematics were statistically indistinguishable. **(B)** Confusion matrices based on the spiking activity of 31 MIP neurons tested with both normal and abducted initial arm postures. Data from trials involving different joint kinematics were not confused by the decoder (off diagonal matrices), implying that MIP activity can distinguish among movements involving different trajectories in joint coordinates.

One plausible interpretation of the findings shown in Figure 8 and of the complex changes in memory tuning of Figure 9 is that changes in activity during the obstacle block partly reflect changes in planned reach trajectories in *intrinsic* (joint/muscle) coordinates. In support of this idea Figure 10 shows example behavioral trajectories (A) and decoding results for 35 neurons (B) under conditions where direct reaches were performed using either naturally assumed initial arm postures (red posture) or involuntarily abducted arm postures (blue posture). Figure 10A shows that endpoint trajectories were similar in these two conditions in terms of their curvature, length and speed maxima (stars). In contrast, the two different starting arm configurations, postural trajectories differed substantially between these two conditions. The decoding analyses (Figure 10B) showed that memory activity from both conditions could accurately predict the target location/final movement endpoint (main diagonal matrices). Moreover, blocks of trials involving the same endpoint kinematics but different joint kinematics were not confused by the decoder (minor diagonal matrices, bottom left and top right). In other words, preparatory activity in MIP easily distinguished not only impending movements to different target locations, but also impending movements to the same target location involving different postural paths.

## Discussion

Here we examined the planning activity of MIP neurons when animals reached directly between two locations in space as well as when they moved between these locations in the presence of an intervening obstacle which they had to avoid. Most neurons changed their firing rates dramatically when moving in the presence of the obstacle, which required changes in both the spatial and temporal aspects of movement. When the obstacle was removed, animals reverted back to their initial behavior and the firing rates of many individual neurons also tended to revert back toward their initial levels. Importantly, despite these changes at the single cell level, the activity of the population accurately predicted the upcoming movement trajectory at both the extrinsic (hand) level and the intrinsic (postural) level. These results suggest that ***reach planning*** activity in the PPC reflects aspects of the impending motion trajectory that an animal will use to move between two points in space. These findings have broad implications for understanding the neural correlates of movement planning in general, as well as for the specific role of the PPC in this process. The findings may also have important implications for the cortical control of prosthetic devices, particularly those devices designed to emulate a humanoid arm.

### Movement planning

Many previous studies have pointed to a role for the PPC in movement planning though its precise role in trajectory planning computations remains unclear. Early studies of LIP and MIP suggest that PPC activity reflects high level motor intentions, i.e., abstract movement plans specifying the spatial goal of movement as well as the effector or effectors (eye, hand, etc.) used to attain that goal (reviewed by Andersen and Buneo, 2002; Andersen and Cui, 2009). More recent studies in both humans and monkeys suggest that reach-related activity in the PPC represents not only the reach goal but also the starting position (Buneo et al., 2002; Beurze et al., 2007; Khan et al., 2007; Chang et al., 2009), information that could be used to calculate a hand-centered movement vector. Studies of dorsal area 5 (area 5d), which lies adjacent to MIP, have also clearly pointed to a strong influence of postural information in cell discharge (Lacquaniti et al., 1995; Scott et al., 1997), and activity starts to increase until the effector is unambiguously specified as arm (Cui and Andersen, 2011). Interestingly, recent anatomical studies have identified proprioceptive pathways to MIP from the dorsal column nuclei and the postcentral somatosensory cortex (Prevosto et al., 2011). Other studies of area 5d indicate, however, that dynamic aspects of movement (i.e., joint torques and/or endpoint forces) may not be as strongly represented in PPC as they are in motor areas of the cortex (Hamel-Paquet et al., 2006). These previous findings, along with those presented here, suggest that the PPC plays a broader role in movement planning than has previously been suggested, and that this role may involve specifying kinematic details of arm motion but not necessarily the forces and torques that give rise to that motion.

The present investigation uncovered a population of cells for which an intrinsic postural signal coexisted with a hand-centered code specifying extrinsic trajectories. For some cells, tuning for target location/endpoint trajectory during the memory period remained invariant to changes in required trajectories; responses of these neurons were typically characterized by simple rate scaling effects with changes in arm posture. Other cells exhibited more *complex* effects of arm posture (i.e., shifts in tuning and gain modulation). Thus, the tuning to impending trajectories in extrinsic and/or intrinsic space suggests that MIP plays a role in mapping movement goals in visual coordinates into corresponding trajectories in extrinsic and/or intrinsic coordinates (i.e., an inverse map). Conversely, the results also indicate that the hand-centered movement vector can conceivably be extracted from postural signals represented in the PPC, and provide information related to the solution of the kinematic redundancy problem for the arm (Buneo and Andersen, 2006). Although the present findings do not indicate the precise nature of this solution at the neural level, it does provide evidence that all the necessary ingredients for a direct transformation between intended hand direction—which we can visually sense—and the corresponding arm posture—which we can kinesthetically sense—coexist in the same population of PPC neurons. Computational models of a direct transformations between these two forms of movement representation have been previously proposed (Torres and Zipser, 2002; Torres and Andersen, 2006; Biess et al., 2007, 2011) which are consistent with the least action principle (Lanczos, 1966; José and Saletan, 1998).

The activity of PPC neurons in this task can also be envisioned as a hybrid representation that embeds a representation of the desired reach vector in the postural representation, the former specified in either eye and/or hand coordinates. This would be a particularly useful representation for obstacle avoidance, as behavioral data suggest that the planning of arm movements around obstacles takes into account not only the physical properties of the obstacles in extrinsic space (Chapman and Goodale, 2008) but also intrinsic factors such as the anisotropic inertial properties of the limb, uncertainty in joint level control or uncertainty associated with proprioceptive input (Sabes and Jordan, 1997). Viewed in this context, the observed enhancement in planning activity during the memory period might then correspond to anticipatory patterns of variability along those dimensions in postural space that are most directly relevant for successfully avoiding the obstacle (i.e., a “sensory prediction”). By contrast, the suppression in planning activity would correspond to anticipatory patterns of variability in other joint angle dimensions (“incidental” to the task) (Torres et al., 2011). We are at present investigating this idea in a similar task where the obstacle lies opposite of the cell's preferred spatial direction.

Although the present findings suggest the PPC is involved in aspects of trajectory planning, several important questions about this process remain unanswered. For example, it remains unclear whether memory-period activity in the PPC reflects the complete details of the trajectory in advance of movement onset, or instead reflects the planning of some critical element of the trajectory, such as its curvature, etc., with the remaining details emerging in real-time during movement execution. It is also possible that activity during this epoch represents in part a sensory memory (or lack thereof) of the obstacle's location (McVea and Pearson, 2009). The current experimental paradigm does not allow us to distinguish among the various possibilities at the neural level. However, an in-depth kinematic analysis of the trajectories during this obstacle avoidance task suggests that spatial aspects of the trajectories are resolved in advance of the movement, while temporal aspects are resolved more gradually (Torres and Andersen, 2006; Torres, 2010). Although it is tempting to speculate that the neural changes evoked with the current paradigm reflect this dissociation, further experiments are required to confirm this hypothesis.

### Different neural populations evolving with different time scales

Several studies have pointed to the existence of different populations of cells within several cortical areas based on analyses of extracellular spike width (Mitchell et al., 2007; Chen et al., 2008; Kaufman et al., 2010; Song and McPeek, 2010). We also found evidence for distinct cell types in our population, one relatively narrow spiking (putative interneurons) that was associated with response enhancement and the other relatively broad spiking (putative pyramidal neurons) that was associated with response suppression. This is reminiscent of previous reports along the “what” pathway, showing that V1 and V4 neurons were modulated by task difficulty and attentional demands, respectively, and that these effects were associated with neurons of different spike width (among other factors) (Mitchell et al., 2007; Chen et al., 2008). Our findings along the “where” pathway are different, however, as they also reflect the process of adaptation and subsequent deadaptation that occurred following exposure to the obstacle. For example, even though the monkeys explicitly observed the removal of the obstacle the activity of most neurons did not immediately revert back to the pattern exhibited before the obstacle was introduced. Instead, this change happened gradually and the rate at which this occurred was highly variable from neuron to neuron. In parallel, arm trajectories continued to change, gradually reverting back to the postural paths recorded in D1 (statistics reported in Torres and Andersen, 2006; Torres, 2010). Thus, we do not believe that patterns of response suppression and enhancement described here during the memory epoch represent only attentional demands or task difficulty. These neural patterns were also related to the complex process of adaptation and deadaptation associated with generating the more curved and temporally complex trajectories along different postural paths that were required for avoiding obstacles (see more on Learning, below).

### Learning

Although animals rapidly adapted the spatial aspects of their movements to the presence of the obstacle in this experiment, changes in temporal dynamics developed more slowly and were more variable, suggesting that animals needed to relearn some aspects of the dynamics on a nearly daily basis. Separate longitudinal analyses of the behavioral data revealed that the bulk of the learning variability came from adjustments in the distance traveled by the hand along the curved path (Torres, 2010). We have shown here that these adjustments were linked to changes in firing rates during the last half of the obstacle block. This suggests that some of the observed changes in PPC activity during obstacle avoidance represent a neural correlate of motor learning. Previous studies in both humans and monkeys have also identified neural correlates of learning in this area. For example, a positron emission topography (PET) employing a prism adaptation paradigm revealed selective activation of the PPC contralateral to the adapted arm, consistent with the learning that accompanies this process (Clower et al., 1996). More recently, Mulliken et al. (2008b) showed strong learning related effects in the PPC during brain control in monkeys, which manifested as an increase in tuning depth of individual neurons, increased coverage of the parameter space and an overall increase in decode performance over a period of several days. The present work further emphasizes the role of the PPC in motor learning and suggests that this learning involves changes in both the spatial and temporal domains. Such involvement in motor learning had not been previously reported in the PPC.

An alternative interpretation of the observed adjustments to the speed profiles is that they reflect the motor system's attempt to recall the most appropriate trajectory representation in space and time that would allow successful movement around the obstacle. The two distinct cell types observed in our population, one relatively narrow spiking (associated with response enhancement) and the other relatively broad spiking (associated with response suppression), could conceivably be a reflection of this process. That is, neurons that show enhanced firing rates during obstacle avoidance could relate to trajectories in space and/or time that are similar (in either their spatial, postural or temporal aspects) to the family of trajectories that are being explored by an animal in a given experimental session. Conversely, broad-spiking neurons could reflect preferred spatio-temporal trajectories that are far removed from the ones being explored, and are therefore suppressed. This mechanism could allow learning to focus on neurons whose preferred trajectories correspond closely to the ones being explored. The present paradigm provides new avenues to systematically investigate these open questions.

The most important aspect of the present findings is that the planning signal of the neurons in this region maintained its predictive, anticipatory power despite the often large fluctuations in neural activity associated with the adaptive process that the animals underwent in real time. This type of adaptive neural representation would be required in any implementation of an error-correction code requiring a reliable reference signal. Thus the PPC appears to be a good candidate for such a reference-guiding signal at both the hand and the arm-postural levels of representation.

### Conflict of interest statement

The authors declare that the research was conducted in the absence of any commercial or financial relationships that could be construed as a potential conflict of interest.
